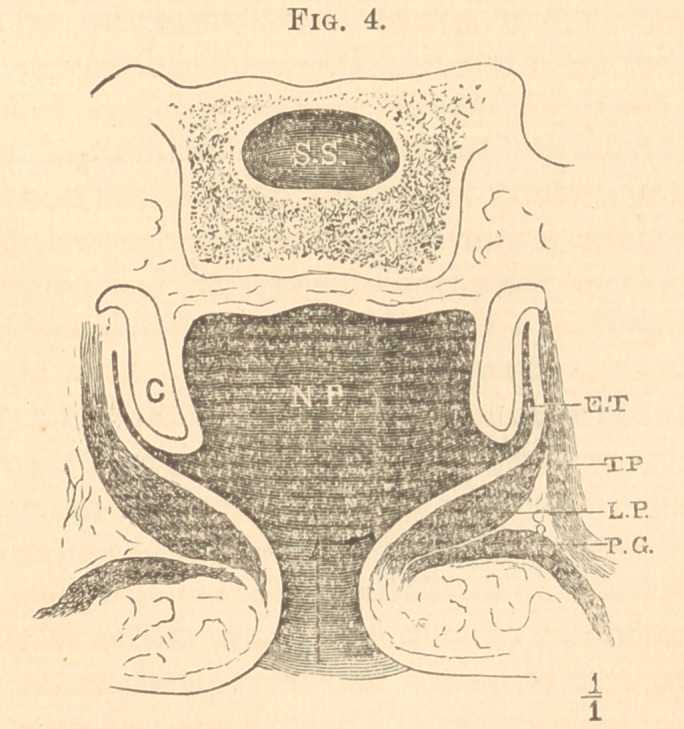# Description of a Specimen of Cleft Palate

**Published:** 1890-04

**Authors:** Johnson Symington

**Affiliations:** Lecturer on Anatomy, Minto House, Edinburgh; Examiner in Anatomy in University of Edinburgh


### DESCRIPTION OF A SPECIMEN OF CLEFT PALATE.[1]

BY JOHNSON SYMINGTON, M.D., F.R.S.E.,

Lecturer on Anatomy, Minto House, Edinburgh; Examiner in Anatomy in University of Edinburgh.

THIS specimen was met with in a male subject, aged seventy, dissected in my rooms last summer session. The cleft was obviously congenital, and extended through both the hard and the soft palates and the right alveolar arch. It opened above into the right nasal cavity.

The upper jaw was practically edentulous, so that it was not possible to determine the relation of the cleft to the incisor teeth. It may be noticed, however, that the cleft passed through the alveolar arch barely a quarter of an inch external to the frænum of the upper lip, so that on the right side there was obviously not room internal to the cleft for more than the central incisor tooth.

Mr. Bowman Macleod kindly made a cast of the deformity for me, and I then froze the specimen, and made a series of transverse vertical sections through the palate, nasal cavities, and maxillary sinuses. Sections of this kind are very useful for the demonstration of the relation of the palate and nasal cavities, and Zuckerkandl[2] has employed this method very extensively for the illustration of diseased conditions of the nasal cavities. I have, however, been unable to find any published drawings of similar sections in cases of cleft palate. Indeed, the illustrations of this condition appear to be practically confined to representatives of the cleft as seen from the mouth. These figures, which are generally diagrammatic, merely represent what can be readily seen on an examination of the deformity in the living body, and give a very incomplete view of the condition of the palate and nasal cavities.

Fig. 1 is a drawing of the cleft in my specimen, as seen from the oral aspect. There is a cicatrix in the upper lip below the right nostril, and it looks as though there had been a hare-lip on that side

---

[1] Read before the Odonto-Chirurgical Society of Scotland, December 12, 1889.

[2] Normale und Pathologische Anatomie der Nasenhöhle. Wien. 1882.

which had been operated on. The anterior part of the alveolar arch to the left of the cleft projects lower down and overlaps somewhat the thickened and warty-like mucous membrane attached to

Fig. 1.

½

the alveolar arch on the right side of the cleft. The left alveolar arch gradually becomes less prominent as it passes backward. The fissure extends through both hard and soft palates, and there are two distinct uvulæ.

Four transverse vertical cuts were made with a saw, so as to divide the specimen into five pieces. The two anterior cuts went through the nasal cavities, and the two posterior ones through the naso-pharynx. The transverse lines on Fig. 1, numbered 1, 2, 3, and 4, indicate the position in which the sections were made.

Fig. 2 is from a tracing of the posterior cut surface of the anterior slab. The ethmoidal sinuses and superior and middle turbinated processes are fairly symmetrical, except that the right middle turbinated process is distinctly smaller than that of the left. The septum nasi passes downward, and slightly to the right, for one and one-fourth inches. At this point it is thickened, and then makes a very marked bend downward and to the left, to join the left palatine process.

It will be seen that the fissure, although opening into the right nostril, is situated to the left of the mesial plane, and the closure of the left nasal cavity is not associated with any marked development of the palatine process on that side, but depends upon the deflection of the septum nasi to the left. The vertical thickness of the left

FIG. 2.

alveolar arch is decidedly greater than that of the right, but it lies farther from the mesial plane. The antrum is larger on the left, LA, than on the right, RA, side. The openings from the antra into the infundibula are anterior to the section, and there are no apertures leading directly from the antra into the middle meatuses.

Fig. 3 shows the posterior surface of the second slab. It will be observed that in this plane the septum has a very prominent ridge projecting from its right side into the space between the superior and middle turbinated processes. Below this ridge the septum inclines downward and slightly to the left. The antrum extends much lower down on the left side than on the right.

Fig. 4 is taken from the posterior surface of the fourth slab. The body of the sphenoid is divided nearly half an inch behind the posterior clinoid processes. The left sphenoidal sinus is opened, but the right one does not extend so far back. The section is a little behind the pterygoid processes, and corresponds to the pharyngeal ends of the Eustachian tubes. Each Eustachian tube is bounded internally and above by its cartilage, the outer wall being membranous. The two halves of the soft palate are of about the same thickness. Below the Eustachian orifices they are about three-quarters of an inch, thick, but become rather thinner as they

FIG. 3.

approach the mesial plane. This section shows extremely well the relations of the palatal muscles. The levator palati forms a well-

FIG. 4.

defined mass of muscular tissues, which lies just beneath the mucous membrane, covering the upper surface of the soft palate. The tensor palati appears as a thin sheet of fibres lying external to the Eustachian tube. On the right side, after removing a little fat, its tendon was easily traced to the hamular process of the internal pterygoid plate. A small bundle of fibres connected internally with the lower part of the levator palati, and passing outward and downward, belongs to the palato-glossus. The section is immediately in front of the tonsils, and consequently anterior to the palato-pharyngeus.

The muscles of the soft palate are separated from the mucous membrane on the oral surface of the palate, by a thick layer of glandular tissue and fat. It is scarcely necessary to point out how clearly this specimen demonstrates the relations of the muscles of the soft palate, as described by Sir William Ferguson. It also shows that the levator palati lies much nearer the upper than the lower surface of the soft palate, and, therefore, can be most readily divided by Ferguson's method.

## Figures and Tables

**Fig. 1. f1:**
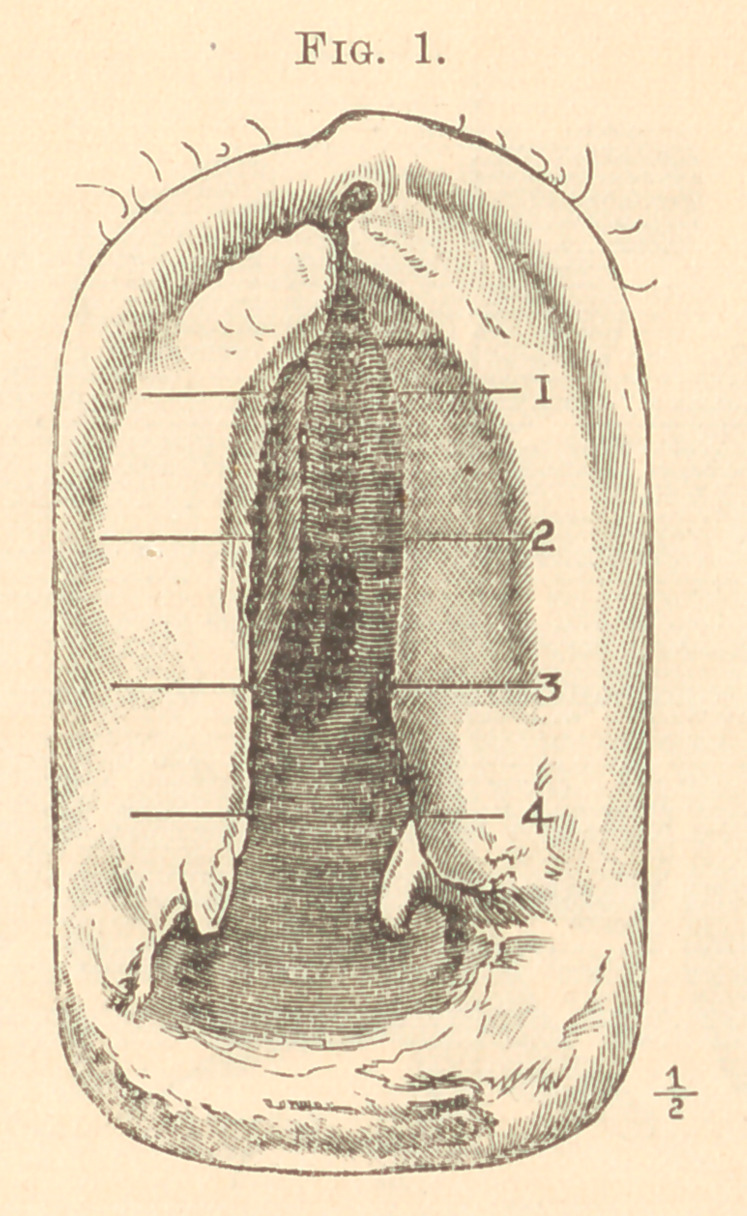


**Fig. 2. f2:**
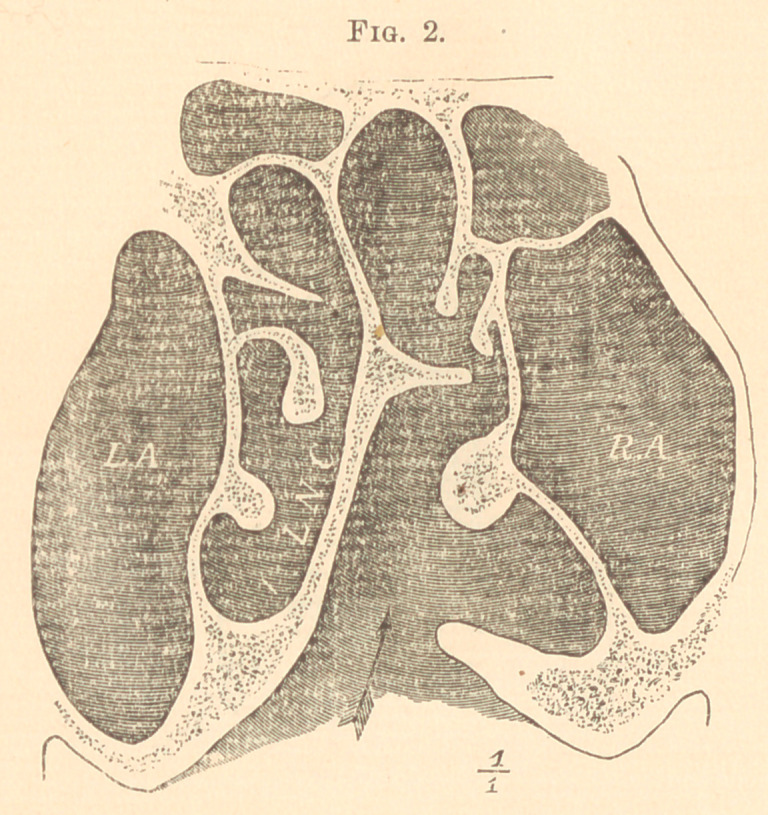


**Fig. 3. f3:**
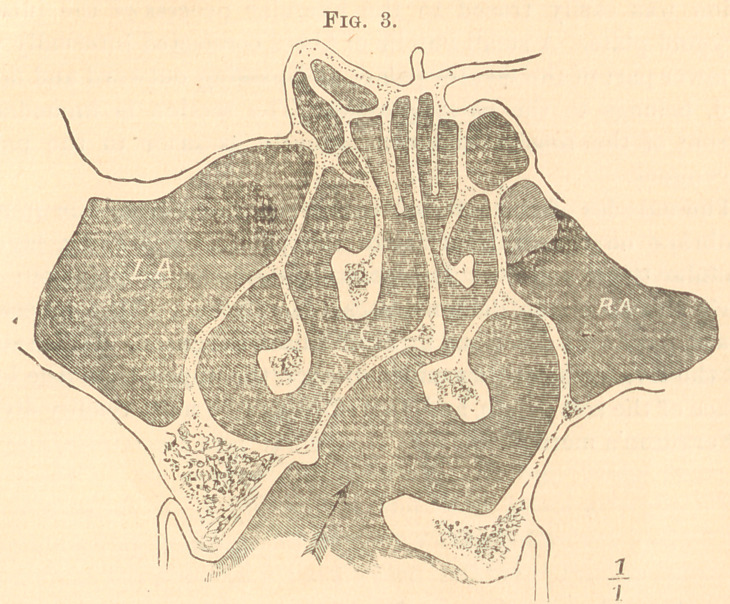


**Fig. 4. f4:**